# Differential Medial Temporal Lobe and Parietal Cortical Contributions to Real-world Autobiographical Episodic and Autobiographical Semantic Memory

**DOI:** 10.1038/s41598-018-24549-y

**Published:** 2018-04-18

**Authors:** Thackery I. Brown, Jesse Rissman, Tiffany E. Chow, Melina R. Uncapher, Anthony D. Wagner

**Affiliations:** 1 School of Psychology, Georgia Institute of Technology, Atlanta, Georgia United States of America; 2 Department of Psychology, University of California, Los Angeles, Los Angeles, California United States of America; 3 Department of Neurology, University of California, San Francisco, San Francisco, California United States of America; 4 Department of Psychology, Stanford University, Stanford, California United States of America; 5 Stanford Neurosciences Institute, Stanford University, Stanford, California United States of America

## Abstract

Autobiographical remembering can depend on two forms of memory: episodic (event) memory and autobiographical semantic memory (remembering personally relevant semantic knowledge, independent of recalling a specific experience). There is debate about the degree to which the neural signals that support episodic recollection relate to or build upon autobiographical semantic remembering. Pooling data from two fMRI studies of memory for real-world personal events, we investigated whether medial temporal lobe (MTL) and parietal subregions contribute to autobiographical episodic and semantic remembering. During scanning, participants made memory judgments about photograph sequences depicting past events from their life or from others’ lives, and indicated whether memory was based on episodic or semantic knowledge. Results revealed several distinct functional patterns: activity in most MTL subregions was selectively associated with autobiographical episodic memory; the hippocampal tail, superior parietal lobule, and intraparietal sulcus were similarly engaged when memory was based on retrieval of an autobiographical episode or autobiographical semantic knowledge; and angular gyrus demonstrated a graded pattern, with activity declining from autobiographical recollection to autobiographical semantic remembering to correct rejections of novel events. Collectively, our data offer insights into MTL and parietal cortex functional organization, and elucidate circuitry that supports different forms of real-world autobiographical memory.

## Introduction

Cognitive science has traditionally divided declarative memory into systems underlying episodic memory (autobiographical events) and general semantic memory, and drawing a contrast between episodic and semantic memory has proven useful for understanding the neural systems that underlie these qualitatively distinct forms of remembering. At the same time, it is critical to appreciate that autobiographical remembering can itself be subdivided into autobiographical episodic (event) memory and autobiographical semantic memory (remembering personally relevant semantic knowledge; also referred to as personal semantics)^1,2^. For example, when we review photos from a recent vacation, this may cue detailed memories for where the photos took place and the sequence of events surrounding the images. Moreover, in addition to or instead of remembering such episodic details, we may remember autobiographical semantic details, such as the fact that we vacation at that spot every summer or recognizing our bicycle leaning against a wall in the images. Autobiographical semantic knowledge is unique to an individual and constitutes an integral component of autobiographical event memory, but, like general semantic memory, it is acontextual and generalizes across distinct episodes from our lives.

There is increasing interest in understanding whether the neural bases of autobiographical episodic and semantic remembering are shared or distinct. In particular, while autobiographical semantics can be experienced independent of recalling a specific experience, there is debate about whether the neural substrates of episodic recollection, including the medial temporal lobe (MTL) and subregions of parietal cortex, contribute to autobiographical semantic remembering. One line of evidence, drawing on foundational fMRI work, suggests differentiation between the circuitry underlying episodic and autobiographical semantic memory^3^. By contrast, a second line of evidence suggests that, depending on how memory is measured, the neural substrate of autobiographical semantics may more closely resemble either that of episodic memory or of general semantic memory. In particular, when remembering personal facts (such as a sibling’s name), autobiographical semantic memory may more closely resemble general semantic memory, whereas autobiographical semantic memory may draw more strongly on circuitry associated with episodic memory when it involves repeated experiences (temporally-extended but non-episodic) or semantic information and concepts that have high personal or emotional significance^1,2,4^. For example, neuropsychological data suggest memory for autobiographical facts (e.g., “I grew up in Vermont”) may be more directly built upon associations between spatio-temporal and perceptual details and, unlike knowledge of traits (“I am short”), appear to rely on the MTL, albeit to a lesser degree than autobiographical recollection^2^. Similarly, parietal subdivisions have highly varied associations with different forms of autobiographical semantics^1^, but as we discuss below, specific subcomponents appear to support functions that span general semantic remembering, autobiographical semantics, and recollective experience. Finally, a third line of evidence, using network-level analyses targeting autobiographical memory, laboratory-based episodic memory, and (non-autobiographical) semantic memory, suggests that a common neural network may underlie the retrieval of declarative memories regardless of memory content^5^.

A growing body of neuroimaging data has characterized the functional contributions of frontoparietal and MTL subregions to episodic retrieval^6–11^. Critically, the specific profile of activation within these regions is diagnostic of the subjective feeling of familiarity for a given retrieval cue versus the recollection of associated contextual details^12–16^. It is now understood that considerable functional heterogeneity within the parietal cortex, as well as in the MTL, maps onto subjective episodic memory states and related computations (such as memory decision uncertainty)^9,17^, and that frontoparietal and MTL activity patterns can be leveraged to classify autobiographical episodic memories based on subjective experience^18^.

Functional differentiation within the MTL may contribute to the different experiences of autobiographical event and autobiographical semantic memory. The hippocampus is known to be critical for episodic memory for recent past events (i.e., for event memories that have not undergone systems consolidation), whereas semantic knowledge (including autobiographical semantics) can be retrieved independent of the hippocampus^13,19–22^. Moreover, along a rostrocaudal gradient, posterior MTL cortex has been linked to scene processing, while anterior MTL cortex has been differentially linked to face, object, and item content representations^23–26^. The collective functional and anatomical data suggest hippocampus and its posterior parahippocampal inputs may play a fundamental role in processing situational information and contexts, linking mnemonic traces to such states^27,28^. Perirhinal cortex, by contrast, has been posited to play a role in semantic coding, including during episodic memory paradigms^29–34^, raising the possibility that content representations in anterior MTL cortex contribute to autobiographical semantic remembering.

Beyond the MTL, recent declarative memory research has sought to delineate the mechanistic significance of functional heterogeneity consistently observed in left posterior parietal cortex (PPC). During episodic retrieval, a parietal “Old/New” effect — differential activation to recognized studied memory probes vs. correct rejection of novel memory lures^15,17,35–37^ that scales with perceived memory strength^9,17^ — is present in the lateral intraparietal sulcus (IPS). By contrast, activation in the superior parietal lobule (SPL) and medial IPS tracks uncertainty about recognition memory decisions^9,17^. Finally, in more ventrolateral PPC — specifically, angular gyrus (AnG) — activity is associated with the experience of episodic recollection (for reviews and theoretical perspectives, see^7,38,39^). Moreover, and critically, AnG activity has also been associated with general semantic processing in non-episodic memory tasks^40–44^, as well as with judgments of whether imagery is self-relevant even when episodic memory is absent^45^. Consequently, a fundamental question is whether the PPC regions that show parietal Old/New, decision uncertainty, and episodic recollection effects also contribute to autobiographical semantic remembering.

One theory of PPC contributions to memory holds that lateral IPS mechanisms accumulate evidence in service of making a mnemonic decision^10,15,46^, and it is presently uncertain whether this function extends to autobiographical semantic decisions and/or laterally into areas such as AnG^9,47^. The mnemonic functions of AnG are particularly relevant for the present work, as extant data suggest that its contributions to declarative memory may generalize beyond episodic recollection (e.g., AnG activation is observed during non-autobiographical semantic retrieval tasks)^40–42^. Accordingly, it is possible that AnG, while often associated with episodic recollection vs. familiarity, could contribute to autobiographical semantic remembering. Moreover, to the extent that semantic knowledge is a component of richer autobiographical event memories, it is possible that autobiographical semantic retrieval may contribute to the amount of convergent information in AnG^14,39^ that underlies autobiographical episodic memory judgments.

Efforts to compare brain activation during retrieval of laboratory-encoded and real-world event memories have documented numerous differences^48–50^, some of which may be attributable to task differences. Studies of autobiographical semantic retrieval are comparatively sparse, with functional associations that – as noted above – vary based on how memory is measured^1,2,4^. Therefore, examining real-world autobiographical semantic and autobiographical event remembering within a single memory decision task presents opportunities for understanding and refining declarative memory boundaries and their underlying neural mechanisms. Gaining a better understanding of the neural substrate of autobiographical semantics is also relevant for challenges in applied neuroscience, such as understanding limitations in the forensic use of EEG or fMRI for probing experiential knowledge. Recent work has underscored both the potential and the limitations of using fMRI to detect the presence and subjective quality of laboratory-based and real-world event memories^18,51,52^. To the extent that autobiographical semantics share a common neural basis with and contribute to autobiographical event memory, the ability to leverage neural measures in order to discriminate memories for personally relevant information from personal event memories may be hampered.

Collectively, extant data suggest that activation profiles across MTL and parietal subregions may reflect retrieval mechanisms that contribute to and distinguish between autobiographical episodic and autobiographical semantic remembering. Using photographs of participants’ everyday personal experiences—captured by wearable cameras—as memory probes during an fMRI-scanned retrieval task, we examined how neural activity during real-world autobiographical episode retrieval relates to activity during retrieval of autobiographical semantic knowledge. We hypothesized that hippocampal recruitment would selectively support autobiographical event retrieval. Consistent with prior literature, autobiographical episodic memory would be further supported by AnG and posterior MTL cortex. We hypothesized that autobiographical semantic remembering would be supported by anterior MTL cortex and AnG due to their functional associations with general semantic processing, as well as item familiarity and recollection, respectively. As such, we predicted that signals in AnG would track event recollection in addition to and beyond the recognition of autobiographical semantic information when episodic memory is absent. It was an open question whether lateral IPS Old/New and SPL/medial IPS decision uncertainty effects are selective to episodic decisions or are also present during semantic remembering.

## Methods

### Participants

We report data from 22 participants (10 women; aged 18–22 years) who participated in either of two closely-matched autobiographical memory camera studies conducted by our group^18,53^. We pooled data across these studies to ensure sufficient statistical power for the present analyses, when considering both participant count and within-participant condition-level trial counts. Specifically, a subset of the 16 participants from each study (32 total) were included in the present set of analyses on the basis of having a sufficient number of trials (5 trial minimum) in all memory conditions of interest (Recollection, Familiarity, Know; described below). We set this threshold prior to data analysis, based on the fact that a subset of participants rarely if ever made responses of a certain type (due to response bias or notably strong or poor memory). Of the 32 total participants: one was excluded due to insufficient Recollection responses, three were excluded due to insufficient Familiarity responses, and five were excluded due to insufficient Know responses; one additional participant was excluded because they made over twice as many incorrect vs. correct Know responses, suggesting they did not use this response category appropriately. In our final sample of 22 participants, the mean trial counts per condition were 37 Recollection hits, 42 Familiarity hits, and 20 Know hits. Only three participants were at our threshold with five trials in a given condition (one participant with five Recollection hits and two participants with five Know hits). We note that, because of fewer test trials in Study 2, participants with these low trial counts were all members of Study 2; importantly, our analyses reported below reveal no significant effects of Study on our critical outcomes.

Written informed consent was obtained from all participants, in accordance with procedures approved by the institutional review board at Stanford University. All methods were performed in accordance with the relevant guidelines and regulations approved by the institutional review board at Stanford University. All participants were right-handed native English speakers, had normal or corrected-to-normal vision, and were prescreened for the presence of medical, neurological, or psychiatric illnesses and use of psychoactive medications.

### Experimental design

The experiment targeted neural correlates of real-world autobiographical memory retrieval using photographs of everyday scenes captured from each participant’s personal experiences using wearable cameras. To provide some control over the nature of the events experienced by our participants, enrollment was restricted to Stanford undergraduate students residing on campus. In both camera studies contributing data to the present analysis, stimuli were collected over an extended period (Study 1^18^: one three-week period of camera wearing followed by a one-week lag prior to the fMRI session; Study 2^53^: three two-week periods, with one two-weeks prior to the fMRI session, one ~3 months prior to fMRI, and one ~6 months prior to fMRI). With the exception of having manipulated memory age in the second study, both datasets were collected following the same overall procedures. To ensure the combined dataset for the present experiment was composed of memories of a comparable age, for Study 2 we selectively analyzed data elicited by photographs collected during the two-week session immediately prior to scanning.

Detailed information about the general experimental design and stimuli can be found in a prior fMRI publication introducing this real-world autobiographical memory paradigm^18^. Briefly, each participant was provided a Vicon Revue digital camera (Vicon Motion Systems Ltd., Oxford, UK) to be worn throughout the day for one or three three-week periods, respectively. The cameras automatically captured 2–10 color photographs per minute during periods of activity. Before the fMRI session, participants had no knowledge of the specific goals of the experiment, but were aware that the images from their cameras would be utilized as stimuli.

For the two studies, we selected 180 (Study 1) and 120 (Study 2) sets of four-photo “event sequences” from the thousands of photos captured by each participant’s camera, to be used as unrehearsed “Own Life” memory probes. Each sequence was composed of four photos depicting the temporal unfolding of a potentially memorable episode from the participant’s day (note: each event sequence spanned up to 5-min in duration). Image content of the selected event sequences varied widely; many sequences contained visible faces, whether of friends, acquaintances, or strangers, as well as personal property and salient environmental features. The research assistants who selected the event sequences were told to think about whether they might be able to remember a given event sequence if it had been from the past month of their own life and they were subsequently shown the four photographs. Many events presented the camera wearer on the move (e.g., walking or biking across campus, hiking a trail, shopping at a mall), or, if stationary, a potentially recognizable event due to the setting (e.g., sitting in a classroom, at a concert, sporting event, or restaurant). Due to the challenges of creating 60 potentially-memorable event sequences per week per participant, we occasionally had to break longer duration events (e.g., a picnic or party) into two or more qualitatively distinct subevents (e.g., arriving at a picnic table and setting up food vs. a later event depicting them playing Frisbee in the park) rather than using multiple repetitive image sequences across days for which the ability to distinguish a given event was judged unlikely (such as frequently repeated sequences of walking down a dormitory hall).

Although it was impossible to avoid the inclusion of multiple similar events (e.g., socializing in the same place with the same group of friends), we embrace this variance as a naturalistic feature of the stimulus set that served to elicit a range of subjective memory retrieval experiences of interest (and as such, enhance the ecological validity of the present experiment). Notably, by virtue of all participants sharing Stanford residency, “Other’s Life” event sequences represented novel memory lures that could share myriad features with Own Life events. Thus, correct rejection of lures stressed the absence of autobiographical memory content.

#### fMRI task design

The two fMRI studies included 300 trials (Study 1) or 360 trials (Study 2) distributed across 10 or 8 scanning runs, respectively. On each fMRI trial, participants were presented with a four-photo event sequence and asked to make a response indicating their memory for that event. Across runs, participants encountered 180 (Study 1) or 120 (Study 2) Own Life trials, and 120 Other’s Life event sequences. Study 2 also incorporated 120 Own Life event sequences that had been explicitly rehearsed during laboratory visits, but these were excluded from fMRI analysis in the present experiment to equate the memory probes underlying Own Life/Other’s Life activation differences (data from the event sequences captured more than one-month prior to the fMRI session were also excluded from the present experiment to equate the age of Own Life memory probes between datasets).

**Figure 1:**
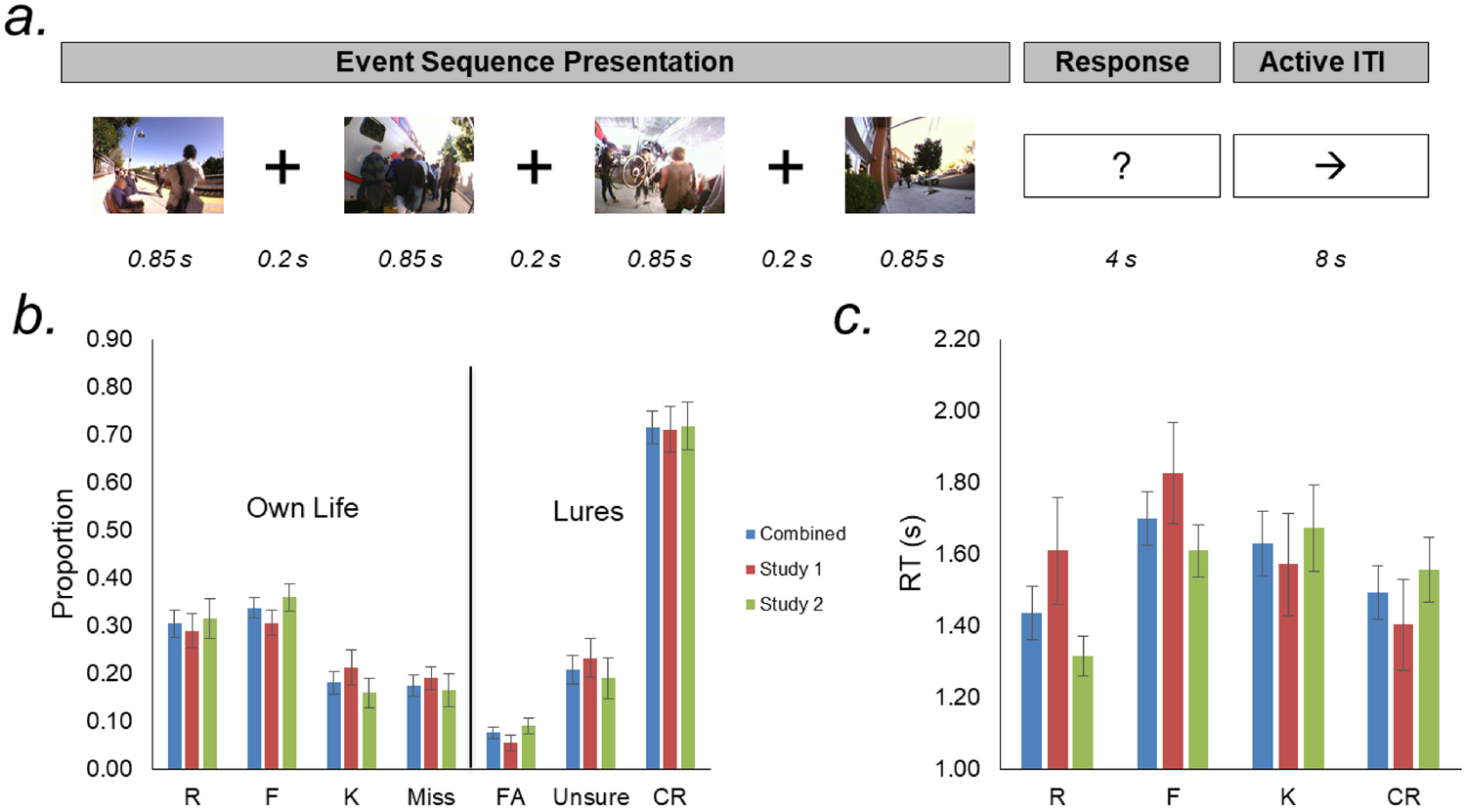
Experimental design and behavioral data. (**a**) On each trial, participants viewed a sequence of 4 photographs depicting the temporal unfolding of an event (note: individuals’ faces and bodies were not blurred in the actual stimulus sets). Immediately thereafter, participants made a button-press response indicating their memory for that event. During the 8 s inter-trial interval, participants were tasked with judging the right/left direction of a series of five arrows. (**b**) Proportional responses for distinct memory categories (R – recollection, F – familiarity, K – know, FA – false alarm, and CR – correct lure rejections). (**c**) Reaction times for memory categories entered into fMRI analysis. F and K responses tended to exhibit the slowest RTs.

The structure of each fMRI trial (Fig. 1a) was as follows: The four constituent photos of an event sequence were sequentially presented for 850 ms each, with a 200 ms central fixation cross appearing between successive photos. After the offset of the fourth photo, a question mark appeared on the screen for 4 s, turning from white to red during the final 1 s to inform participants of the impending deadline for them to make a response. The response period required participants to depress one of eight buttons indicating their level of memory for the event sequence (see below). Participants performed an active baseline task during the 8 s intertrial interval (ITI), to limit mind-wandering or rumination (for additional details, see^18^). The timing of stimulus presentation and response collection was controlled using the Psychophysics Toolbox^54^ in MATLAB (The MathWorks, Natick, MA). Stimuli were projected onto a screen against an isoluminant gray background and viewed through a mirror.

Given the potential subtlety of the distinctions between different subjective memory responses in our study, immediately before the scanning session, participants were provided with and guided through detailed written instructions (see Supplement) that emphasized the critical distinctions between five qualitatively distinct memory experiences:

- Recollected (R): *The participant is able to recollect specific episodic details of the experience* depicted in the photos. Participants could indicate *strong* or *moderate* recollection.
- Familiar (F): *The specific episode seems familiar to the participant*. Participants could indicate *strong* or *moderate* episodic familiarity.
- Know but not familiar (K): *The participant knows that this was their experience based on content in the images, but the specific episode depicted does not seem familiar*. Know responses were designed to assess the recognition of autobiographical semantic information in the absence of episodic memory.
- Unsure: *The participant is unsure whether this was their experience*.
- Not yours: *The participant indicates they did not have this experience* (i.e., that it must have come from another participant’s camera). Participants could indicate *strong* or *moderate* confidence with this novelty judgment.

As detailed in the Supplement, the instructions included specific language designed to ensure that participants were clear on the conceptual differences between recollection, familiarity, and knowing, using specific examples to illustrate these qualitatively different memory states. As part of the instructions procedure, the experimenter also verbally confirmed the participant’s understanding of the instructions and administered a practice version of the retrieval task. The practice version consisted of 12 trials — six Own Life event sequences drawn from surplus photo sequences and six Other’s Life event sequences also drawn from surplus sequences; this task provided participants the opportunity to practice the application of these response types. In the scanner, responses, including confidence levels, were made on two MR-compatible button boxes, one held in each hand. Participants practiced the response options and the use of the button boxes prior to beginning the memory test^18^. In a post-scan interview, participants were asked about their strategies and the most useful stimulus features for their memory decisions (see Supplement).

#### fMRI Data Acquisition

Whole-brain imaging was conducted on 3 T GE Signa and Discovery MR750 systems (GE Healthcare Systems, Milwaukee, WI). Functional images were collected using a T2*-weighted 2-D gradient-echo spiral-in/out pulse sequence (repetition time [TR] = 2.0 s, echo time = 30 ms, flip angle = 75°). Each functional volume consisted of 3.44 × 3.44 × 3.8 mm (Study 1) or 3.28 × 3.28 × 3.3 mm (Study 2) voxels, with slices acquired parallel to the AC–PC plane. The six initial volumes from each run were discarded to allow for T1 equilibration. To aid with spatial registration, anatomical images coplanar with the functional data were collected using a T2-weighted flow-compensated spin-echo sequence, and a T1-weighted whole-brain spoiled gradient recalled (SPGR) 3-D anatomical image (voxel size = 0.86 × 0.86 × 1.0 mm or 0.86 × 0.86 × 0.9 mm).

### fMRI Data Preprocessing

Functional images were preprocessed using SPM5 (www.fil.ion.ucl.ac.uk/spm). Functional images were corrected for differences in slice acquisition timing, followed by motion correction using a two-pass six-parameter rigid-body realignment procedure. The T2-weighted coplanar anatomical image was then coregistered to the mean functional image, and the T1-weighted whole-brain SPGR image was in turn coregistered to the T2-weighted image. The SPGR image was then segmented by tissue type, and the gray matter image was warped to a gray matter template image in Montreal Neurological Institute space. The resulting nonlinear transformation parameters were applied to all functional images, which were then resampled into 3 mm isotropic voxels and smoothed with an 8 mm FWHM kernel.

### fMRI Analysis

Data were entered into 1^st^-level modeling in SPM. Each autobiographical memory event decision was modeled as a 4 s “boxcar”, and convolved with the canonical hemodynamic response function. For the present experiment, there were four critical conditions of interest: successful episodic recollection collapsed across confidence (R hits), episodic familiarity collapsed across confidence (F hits), know, corresponding to autobiographical semantic remembering (K hits); and correct rejection of novel memory lures collapsed across confidence (CR). Events from these conditions were binned into separate regressors, as were memory conditions of non-interest (false alarms, “unsure” responses, and misses). For the second dataset in which memory age and explicit rehearsal of events were manipulated, regressors for different memory response events were further split out along these dimensions. However, as noted above, the present experiment selectively analyzed data from the most-recent, unrehearsed events, which allowed us to combine these data with the data from the first dataset for 2^nd^-level analysis. Importantly, to ensure that there were sufficient trial counts (at least 5) underlying activity comparisons between different subjective memory states, we collapsed across data from different confidence levels (high and moderate) at the 1^st^-level before activity estimates were carried to 2^nd^-level analysis. Regressors modeling movement parameters estimated during realignment and session effects were included in the 1^st^-level models as nuisance factors. An AR(1) model was used to account for serial autocorrelations. GLM parameters were estimated with classical (restricted maximum likelihood) algorithms. Linear contrasts of the resulting parameter estimates were used to investigate and test effects of interest.

Given our strong *a priori* interests in examining functional differentiation within lateral PPC and MTL subregions that contribute to real-world autobiographical remembering, our primary analyses targeted three functional regions of interest (ROIs) in left PPC (AnG, SPL, and lateral IPS; independently defined using data from a separate study of PPC retrieval effects)^17^, and bilateral anatomically defined ROIs within the MTL (hippocampal head, body, and tail; perirhinal cortex - PRC; parahippocampal cortex - PHC). Anatomical ROIs were manually traced using the ITK-SNAP software package (http://www.itksnap.org)^55^ using established procedures^25,56–60^. Using parameter estimates extracted from these ROIs, we examined their functional activation profiles across the autobiographical episodic and semantic remembering conditions. To do so, we implemented two analytic stages using linear mixed effects (LME) modeling in R^61^ (which accommodated unequal sample sizes for the two datasets [included as a factor], and allowed us to maximize the random effect structure while treating participant as a random intercept).

First, we conducted three omnibus LME analyses testing for activation differences between R-F-K-CR conditions across parietal ROIs, across anterior-posterior subdivisions of the hippocampus, and across anterior-posterior subdivisions of MTL cortex (PRC and PHC). These analyses tested for 1) functional sensitivity to memory type, and 2) significant interactions between memory type and neural subregion (as well as interactions with dataset).

Second, given evidence for widespread differences between memory hits and CRs, we conducted separate LME analyses targeting the R-F-K conditions, excluding CRs, within each ROI. This enabled us to test for significant effects of memory type than cannot by driven by CRs, which addressed whether activation profiles within individual subregions were sensitive to different subjective experiences of autobiographical remembering.

Finally, given that the ROI analyses revealed at least four distinct memory-related activation profiles across R-F-K-CR conditions (see below), we conducted exploratory whole-brain voxel-level analyses testing for additional regions with parallel response properties. Motivated by the functional profiles within our a priori ROIs, 1^st^-level contrasts were created in SPM to reveal regions that (1) specifically tracked the strong mnemonic experience of recollection (“R-only” contrast: 3, −1, −1, −1), (2) tracked the presence of episodic memory, regardless of strength (“Episodic-only”: 1, 1, −1, −1), (3) exhibited a non-specific Old/New effect across subjective autobiographical memory experiences (“Memory”: 1, 1, 1, −3), or (4) exhibited a continuous positive linear relationship across memory response types (“Continuous”: 3, 1, −1, −3). Individual participants’ data were then entered into 2^nd^-level analyses using one-sample *t*-tests against zero (treating participant as a random effect and including dataset as a covariate of non-interest). Because effects identified by these different models would not be completely independent, we masked each model to exclude voxels in which pairwise differences between conditions violated the specific response profile being tested (e.g., voxels were excluded from the Memory analysis if they exhibited evidence of an R-F, R-K, *or* F-K difference). To distinguish a Continuous response profile from that of each of the other models, the Continuous model was tested with an inclusive mask of the intersection between R-F, F-K, and K-CR activity differences. A liberal threshold of *p* < 0.1 was applied to all pairwise condition differences used for generating these masks (ensuring there was no evidence of one response profile in the statistical map of another). The resulting group-level statistical maps were corrected for multiple comparisons with a voxel-wise FDR of *p* < 0.05 and a minimum cluster size of 15 voxels. To visualize the profile of the neural circuitry underlying different subjective memory states in our study, we also conducted 2^nd^-level analyses examining the simple effects contrasts of R, F, and K conditions against CRs.

### Data availability

Reported data are archived on the Stanford Neuroscience Institute server. The data are available on the Stanford Digital Repository. There, the data are freely available under the Creative Commons Attribution (CC BY) license at https://purl.stanford.edu/pm789zp5711.

## Results

### Behavioral Results

At test, participants were asked to differentiate Own Life from Other’s Life events (lures). Figure 1b,c depicts the full distribution of proportional behavioral responses, along with the mean RT associated with the target R, F, K, and CR conditions included in the fMRI analyses.

Overall, the mean hit rate was 0.82, with participants responding unsure or “new” for Own Life stimuli on 0.18 of trials. The mean false alarm rates were extremely low, with participants incorrectly identifying lures as being from their own life on 0.08 of trials (mean ± SEM d’ = 2.55 ± 0.12). Because K responses were of particular interest, but were the least frequently selected response for Own Life stimuli, we subsequently computed d’ for autobiographical semantics to confirm above chance performance. To do so, we used corrected K hit and false alarm rates conditioned on the cumulative probability of “superseding” response options - that is, based on an assumed decision-tree in which K is a distinct memory type reported in the absence of an episodic (R and F) memory trace (e.g., IRK procedure)^62^. For autobiographical semantic responses, participants exhibited strong discriminability (mean ± SEM d’ = 1.96 ± 0.13).

Assessment of participants’ responses on a post-scan questionnaire—which should be interpreted with some caution due to their retrospective nature—suggest that autobiographical semantic details, such as people and items (e.g., “bike handles, laptops, teachers, friends”), were an important factor for participants’ memory decisions, particularly deciding whether a memory was Own Life or Other’s Life. Some participants suggested that successful recovery of event memory may have built upon initial recognition of autobiographical semantic information (see Supplement for more details).

Using LME modeling, we confirmed that the proportional response profile across our target conditions (R, F, K, and CR) did not differ between participants from the two datasets (memory type*study interaction *F*_(3,80)_ = 0.64, *p* = 0.59). Contrasts comparing datasets within conditions confirmed that proportional responses within individual memory response types did not differ between datasets (all pairwise *p*s > 0.35).

LME analysis of the R-F-K-CR memory conditions entered into fMRI analysis demonstrated that reaction times significantly differed across memory types (*F*_(3,60)_ = 4.83, *p* = 0.004), and did not differ between datasets (*F*_(1,20)_ = 0.27, *p* = 0.61), although there was a significant memory type*study interaction (*F*_(3,60)_ = 4.11, *p* = 0.01). Contrasts comparing datasets within conditions revealed that this reflected a tendency towards longer RTs for R and F responses in Study 1 (Effect of Study R: *p* = 0.06; F: *p* = 0.17). Consistent with our prior work^17,18^, across datasets F and K responses tended to be the slowest (F > R: *p* = 3.43 × 10^−5^; F > CR: *p* = 0.02; K > R: *p* = 0.03; K > CR: *p* = 0.16). The RTs for F versus K, and for R versus CR did not significantly differ (all *p*s > 0.46).

### fMRI Results

Results (detailed below) revealed that activity in most MTL subdivisions was specifically associated with autobiographical episode recognition. By contrast, the hippocampal tail, SPL, and lateral IPS were similarly engaged when either an autobiographical event memory or autobiographical semantic memory trace was present. Finally, AnG showed a graded response, being recruited during autobiographical semantic remembering, with activity further increasing for autobiographical episodic recollection. In all hippocampal, MTL cortex, and parietal subdivision analyses, there were no significant interactions between the effect of memory type and the dataset factor.

#### Omnibus LME analyses

**Figure 2:**
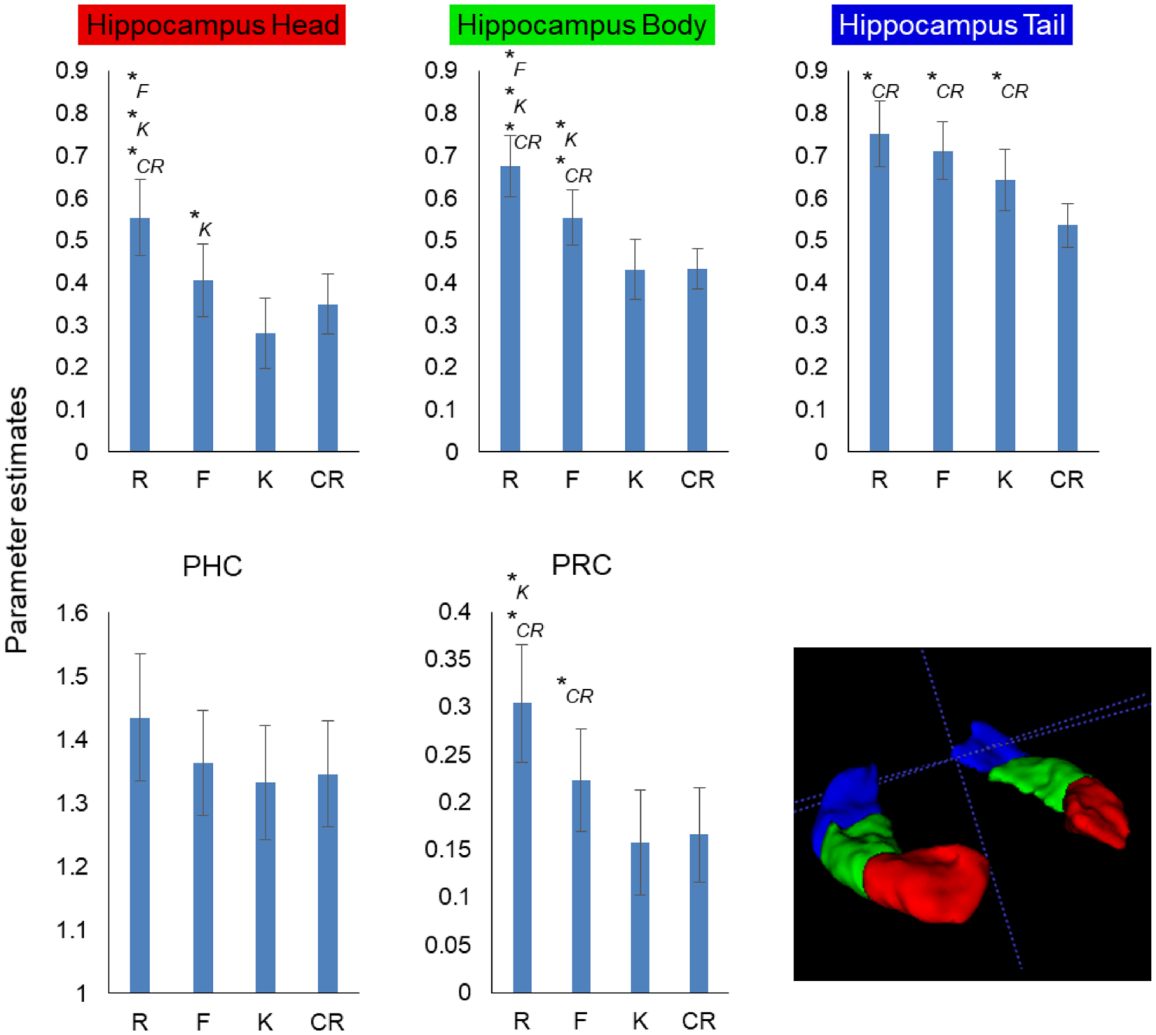
Targeted hippocampus and MTL cortex ROI analysis. Anterior MTL (hippocampus head, body, and PRC) exhibited signal that signaled episodic memory. By contrast the hippocampal tail exhibited a non-specific Old/New effect relative to CR. PHC was characterized by robust above-baseline signal for probe scenes that was not sensitive to memory content. The episodic vs. non-specific Old/New differences along the rostro-caudal axis of the hippocampus were reflected in a significant subregion*memory type interaction. *Indicates condition against which there is a significant simple effects contrast.

LME analysis across anterior-posterior hippocampal subdivisions (with a random intercept of participant and random slopes for subregion and memory type) revealed a significant effect of memory type (*F*_(3,20)_ = 6.16, *p* = 0.004), of subdivision (*F*_(2,20)_ = 13.20, *p* = 0.0002), and a memory type*subdivision interaction (*F*_(6,120)_ = 10.04, *p* = 5.61 × 10^−9^), indicating that the response profile across the different memory conditions differed from anterior to posterior hippocampus (Fig. 2).

LME analysis across anterior-posterior MTL cortical subdivisions revealed a main effect of subregion (*F*_(1,20)_ = 436.05, *p* = 4.89 × 10^−15^), but no effect of memory type (*F*_(3,20)_ = 2.05, *p* = 0.14), and no memory type*subdivision interaction (*F*_(3,60)_ = 1.00, *p* = 0.40) (Fig. 2).

**Figure 3:**
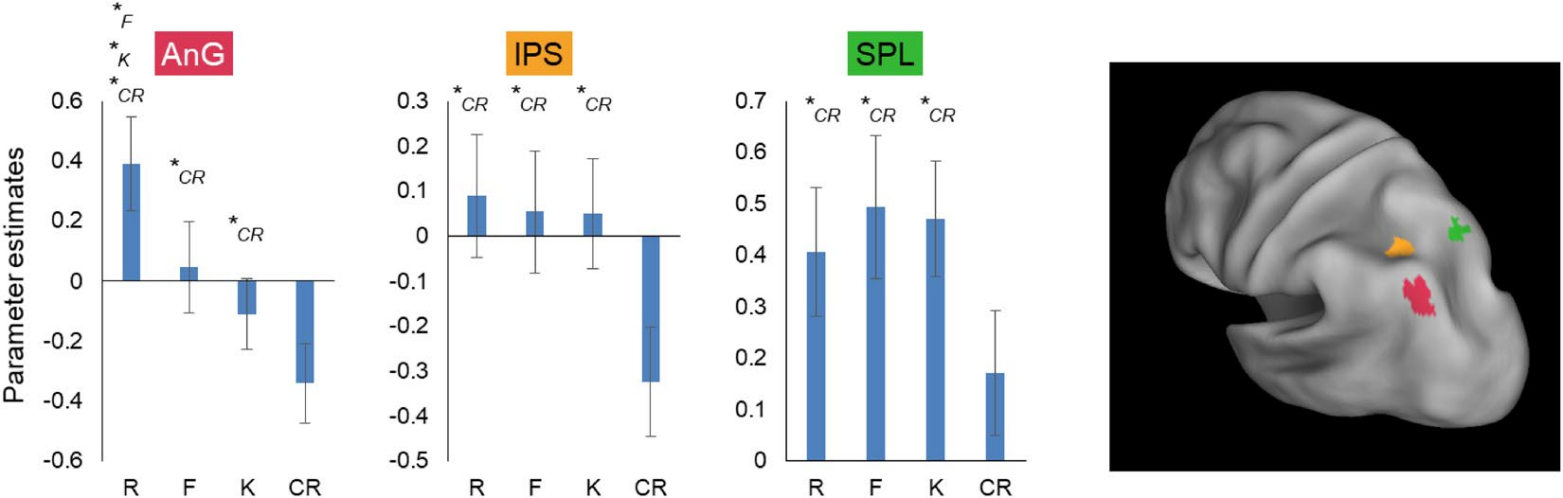
Targeted parietal episodic memory ROI analysis. Angular gyrus exhibited a continuous activation profile that distinguished Autobiographical Semantics (K) from both CR and episodic recollection (R). By contrast IPS and SPL exhibited a non-specific Old/New effect relative to CR. The continuous vs. non-specific Old/New differences within left PPC were reflected in a significant region*memory type interaction. *Indicates condition against which there is a significant simple effects contrast.

LME analysis across parietal ROI revealed a significant effect of memory type (*F*_(3,19.99)_ = 30.86, *p* = 1.06 × 10^−7^), of subregion (*F*_(2,20)_ = 10.44, *p* = 0.0008), and a memory type*subregion interaction (*F*_(6,119.97)_ = 11.45, *p* = 4.29 × 10^−10^), indicating that the response profile across the different memory conditions differed across left parietal subregions (Fig. 3).

Pairwise simple effects (summarized in Table 1), exploring the contribution of CRs to the main effects of memory type, demonstrated widespread activation differences between memory hits and CRs. Within parietal cortex, all subregions distinguished all memory types from CRs. Within the hippocampus, the tail distinguished all memory types from CRs, while activity in the body and head selectively distinguished autobiographical recollection from CRs. While there was no significant memory type*subregion interaction within MTL cortex, only perirhinal cortex distinguished memory hits from CRs, and then only for autobiographical episodic memory responses.

**Table 1:** Paired t-tests between individual memory conditions.

	R>F	R>K	F>K
**Hippocampus**			
Head	**t = 3.17, p = 0.005**	**t = 4.11, p = 0.0005**	**t = 2.51, p = 0.02**
Body	**t = 2.87, p = 0.009**	**t = 4.78, p = 0.0001**	**t = 2.75, p = 0.01**
Tail	t = 1.00, p = 0.33	t = 1.96, p = 0.06	t = 1.48, p = 0.15
**MTL cortex**			
Perirhinal	t = 1.94, p = 0.07	**t** = **2.40, p = 0.03**	t = 1.71, p = 0.10
Parahippocampal	t = 1.54, p = 0.14	t = 1.78, p = 0.09	t = 0.58, p = 0.57
**Parietal cortex**			
Angular gyrus	**t = 3.27, p = 0.004**	**t = 3.79, p = 0.001**	t = 1.41, p = 0.17
Intraparietal sulcus (lateral)	t = 0.52, p = 0.61	t = 0.55, p = 0.59	t = 0.05, p = 0.96
Superior parietal lobule	t = −1.07, p = 0.30	t = −0.89, p = 0.38	t = 0.28, p = 0.78
	**R>CR**	**F>CR**	**K>CR**
**Hippocampus**			
Head	**t = 3.38, p = 0.003**	t = 1.26, p = 0.22	t = −1.66, p = 0.11
Body	**t = 5.05, p = 5.35e-05**	**t = 3.36, p = 0.003**	t = −0.05, p = 0.96
Tail	**t = 4.35, p = 0.0003**	**t = 5.40, p = 2.33e-05**	**t = 2.94, p = 0.008**
**MTL cortex**			
Perirhinal	**t = 3.06, p = 0.006**	**t = 2.47, p = 0.02**	t = −0.24, p = 0.81
Parahippocampal	t = 1.83, p = 0.08	t = 0.45, p = 0.66	t = −0.36, p = 0.72
**Parietal cortex**			
Angular gyrus	**t = 5.94, p = 6.82e-06**	**t = 5.56, p = 1.63e-05**	**t = 2.74, p = 0.01**
Intraparietal sulcus (lateral)	**t = 6.11, p = 4.66e-06**	**t = 5.28, p = 3.14e-05**	**t = 5.30, p = 2.94e-05**
Superior parietal lobule	**t = 4.30, p = 0.0003**	**t = 3.58, p = 0.002**	**t = 3.77, p = 0.001**

Significant differences highlighted in bold.

#### Subdivision LME analyses of subjective memory experience (R-F-K, excluding CRs)

Within the hippocampus, anterior subdivisions only responded to different degrees of event memory, whereas the hippocampal tail exhibited a non-specific Old/New memory response that encompassed all subjective autobiographical memory types including autobiographical semantics (Fig. 2). Specifically, in the hippocampal head, there was a significant effect of memory type (*F*_(2,40)_ = 11.28, *p* = 0.0001), with activity significantly distinguishing R, F, and K responses (Table 1); the hippocampal body exhibited a similar response profile (*F*_(2,40)_ = 13.54, *p* = 3.34 × 10^−5^). By contrast, in the hippocampal tail, there was no effect of subjective memory type (when excluding CRs) on activity (*F*_(2,40)_ = 2.91, *p* = 0.07), although the trend reflected a quantitative difference between autobiographical episodic recollection and autobiographical semantic remembering.

Within MTL cortex, there was no significant omnibus memory type*subregion interaction. However, while PHC did not exhibit significant differential memory-related signals (main effect of memory: *F*_(2,40)_ = 1.81, *p* = 0.18; all simple effects non-significant), PRC exhibited a significant effect of memory type (when excluding CRs) (*F*_(2,40)_ = 4.29, *p* = 0.02), with activity significantly distinguishing R and K responses (Table 1).

Within parietal cortex, the significant omnibus LME memory type*subregion interaction was reflected in a continuous positive linear relationship across memory response types in AnG, and non-specific autobiographical memory Old/New signals in the lateral IPS and SPL (Fig. 3): Specifically, in AnG there was a significant effect of memory type (*F*_(2,40)_ = 12.37, *p* = 6.59 × 10^−5^), with activity significantly distinguishing R from K responses (Table 1), whereas in lateral IPS and SPL the effects of memory type (when excluding CRs) were non-significant (*F*_(2,40)_ = 0.23, *p* = 0.79 and *F*_(2,40)_ = 0.86 *p* = 0.43, respectively).

### Exploratory voxel-level analysis

**Figure 4:**
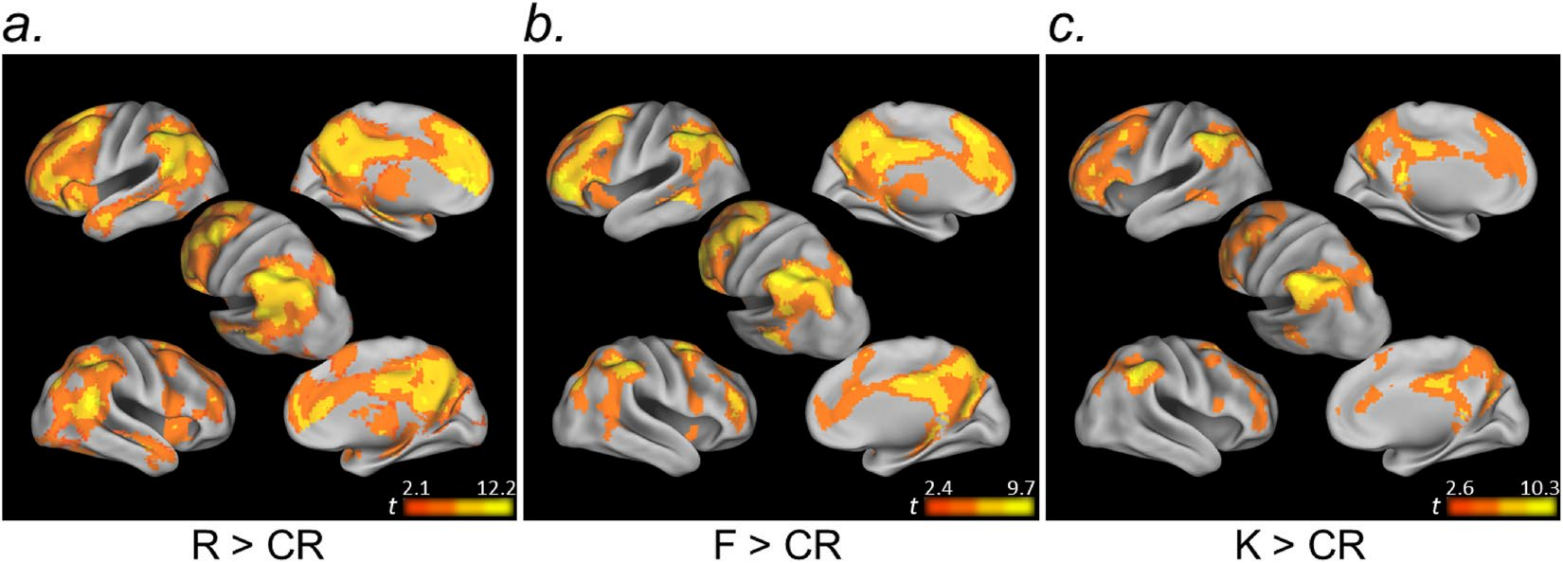
Exploratory voxel-level simple effects analysis. Voxel-level contrasts revealed a qualitatively similar neural circuitry underlying different subjective autobiographical memory experiences (R, F, K) relative to correctly rejected novel memory lures (CR). All maps are thresholded at *p* < 0.05_FDR_, k = 15.

First, voxel-level simple effects analyses revealed a markedly similar neural circuitry underlying different subjective autobiographical memory experiences. Autobiographical semantic recognition was characterized by more focal activity in a qualitatively similar frontal-parietal-MTL network to that engaged during autobiographical event memory in our task (Fig. 4).

Turning to our primary question, R-only, Episodic-only, Memory, and Continuous contrast models of R-F-K-CR activity were associated with distinct functional circuitry within frontoparietal cortex.

1. Consistent with our ROI analyses, activity in bilateral entorhinal/perirhinal cortex, extending into the hippocampal head and amygdala, was selectively associated with recollection. Within parietal cortex, recollection was associated with ventral clusters in angular gyrus (Fig. 5a).

**Figure 5:**
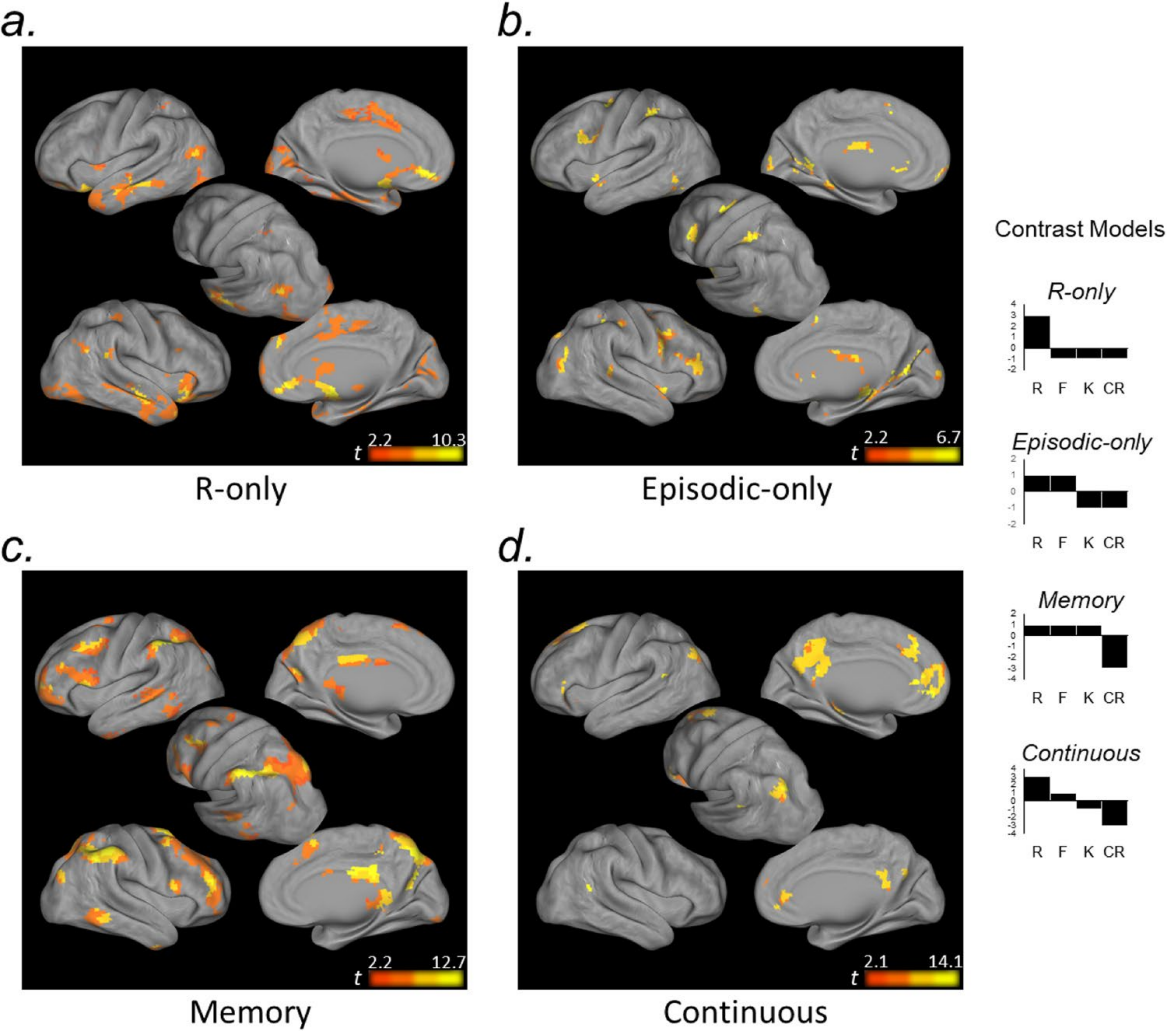
Exploratory voxel-level memory response profile analysis. Voxel-level contrasts illustrating regional response profiles across autobiographical memory conditions (R, F, K, CR). (**a**) Anterior MTL and ventral AnG were associated with recollection. (**b**) Anterior medial IPS, and posterior SFS and IFS were associated with episodic memory retrieval. (**c**) Dorsal PPC and supramarginal gyrus, and cognitive control regions of prefrontal cortex, were associated with non-specific Old/New effects across autobiographical episodic and semantic memory. (**d**) Dorsal AnG, as well as precuneus and medial prefrontal cortex, were associated with a continuous function across R-F-K-CR responses. All maps are thresholded at *p* < 0.05_FDR_, k = 15.
2. The Episodic-only response profile was reflected in posterior frontal and anterior parietal components associated with the dorsal attention network^63^. Additionally, focal bilateral clusters in posterior parahippocampal cortex (and the right hippocampal body [xyz: 24, −28, 10; *t*_(21)_ = 4.66]; not depicted on surface map) tracked episodic memory regardless of memory strength (Fig. 5b).
3. SPL, lateral IPS, the supramarginal gyrus were similarly engaged when memory was based on autobiographical event and autobiographical semantic knowledge (Memory response profile. The Memory response profile was further associated with prefrontal cognitive control circuitry^63^ (Fig. 5c) and posterior-most hippocampal tail (not depicted on surface map; left/right xyz: −21, −40, −4/21, −37, −1; left/right *t*_(21)_ = 5.07/5.42).
4. Lastly, a Continuous response profile was strongly associated with the apex of AnG, and a large extent of the precuneus and retrosplenial cortex, as well as medial prefrontal cortex (Fig. 5d). This profile was also reflected in a cluster in the left hippocampal body (xyz: −27, −28, −13; *t*_(21)_ = 6.85).

## Discussion

Autobiographical remembering can depend on two forms of memory: episodic (event) memory and autobiographical semantic memory (remembering personally relevant semantic knowledge, independent of recalling a specific experience). There is debate about whether the neural signals that support episodic recollection relate to or build upon autobiographical semantic remembering. More broadly, understanding functional heterogeneity within MTL and frontoparietal circuitry is of great significance for theories of long-term memory.

Our experiment utilized wearable camera technology^64^ to enable us to catalog a broad set of potentially memorable events from participants’ daily lives, and to subsequently examine the neural correlates of different forms of real-world autobiographical memory for stimuli drawn from these events. This approach has been used previously to demonstrate that recollection of prior experiences is neurally dissociable from familiarity for life events^65^. Interestingly, in contrast to our findings, this prior study did not reveal sensitivity in the hippocampus to recollection versus familiarity, but implicated both posterior and anterior MTL cortex in familiarity. However, that study employed single photographs as event memory cues and was characterized by a relatively small sample size (N = 10), making it difficult to interpret these between-study differences. More broadly, the use of images from individuals’ lives has proven to be a powerful tool for understanding the neural bases of autobiographical memories and how factors, such as the age of the memory, can influence their underpinnings (e.g., the use of family photographs has revealed that the hippocampus is sensitive to the vividness of autobiographical memories, while areas such as retrosplenial cortex are preferentially engaged for more recent experiences)^66^. Our wearable camera approach presents an opportunity for participants to recognize, relive, or reject candidate memories from the first-person perspective in which they would have experienced those events in their life. While this approach sacrifices some of the control afforded by traditional laboratory-based memory experiments over what stimuli were encoded (for example, it is particularly challenging to know which specific stimulus features gave rise to one subjective memory response versus another), we gained ecological validity through the ability to probe memories for real-world autobiographical events and measure the associated brain activity.

Our results offer several important advances. First, our exploratory voxel-level analysis revealed that relative to episodic recollection, a qualitatively similar, but more restricted, collection of regions was associated with autobiographical recognition based on autobiographical semantic memory (when contrasted against correct rejections). We did not observe any region that was more strongly recruited during autobiographical semantic than autobiographical episodic remembering. One possibility is that, for some participants or for some trials, Know responses reflected very weak familiarity. Although this possibility is difficult to definitively rule out, we note that our participants were provided with highly detailed instructions and fine-grained response options, and all behavioral and self-report indications suggest that participants understood and utilized autobiographical semantic information as a part of their memory decision process. As such, we believe this finding is relevant to the observation that the neural circuitry underlying autobiographical semantic memory appears to more closely resemble that of episodic memory when it is based on temporally-extended sequences (e.g., highly stereotyped repeated events from one’s life) or personally significant concepts or stimuli, as opposed to autobiographical facts (e.g., a sibling’s name) (for review, see^1^). In our study, recognition of personal property, personally-relevant scenery^45^, and acquaintances, rather than – for example – personal traits, was the basis for Know judgments, and the use of naturalistic scene sequence memory probes may favor autobiographical semantic memory judgments that are based on a complex associative structure even in the absence of episodic remembering.

However, our targeted ROI analyses revealed significant functional heterogeneity within the hippocampus, MTL cortex, and PPC that also enables differentiation of autobiographical episodic memory from autobiographical semantic memory, in addition to differentiation of both memory types from lures. Notably, within both the hippocampus and MTL cortex, activity in anterior subdivisions (the head and PRC) was selectively associated with autobiographical episodic memory, particularly the experience of recollection. By contrast, the posterior parahippocampal cortex ROI did not differentiate scene sequences regardless of memory type. Moreover, activity in the hippocampal tail was associated with autobiographical memory regardless of whether it was based on episodic or semantic remembering. This finding indicates that divergent response profiles across the hippocampus and MTL cortex carry information that can distinguish autobiographical episodic memory, autobiographical semantic memory, and the rejection of novel scene lures.

These findings also notably diverge from prior observations of anterior-posterior functional differentiation within the MTL. Although PRC has been associated with semantic coding, including during episodic memory paradigms^29–34^, we did not find evidence that it contributes to autobiographical semantic remembering in our study. Moreover, the literature has predominantly associated posterior hippocampus and MTL cortex with processing situational information and contexts, linking mnemonic traces to such states^27,28^; paralleling strong evidence for different content representations along the anterior-posterior extent of the MTL^23–26^, some researchers have argued that the rostrocaudal extent of the MTL maps onto distinct experiences of episodic recollection (posterior) and item familiarity (anterior)^13^. Our present observations indicate that when measures of autobiographical remembering are broken down into gradations in episodic memory strength, along with event recognition based on autobiographical semantic knowledge, anterior hippocampal and MTL cortex subdivisions more selectively track episodic remembering. In our study, it may be that real-world autobiographical recollection, more so than autobiographical semantic memory and novel scene lures, is characterized by additional processing of items present in scene cues (or brought to mind through pattern completion) in PRC. These findings may also relate to the enriched, temporally-extended nature of the real-world memory cues and the reconstruction of memory narratives from these sequences during recollection. Indeed, convergent evidence suggests that anterior hippocampus may be integral to imagining events and engaging in recollection-based construction of spatially coherent representations^67^. Such findings underscore the importance of continued research aimed at bridging different theories of functional specialization within the hippocampus and surrounding MTL.

Our data also highlight functional differentiation within PPC that relates to different experiences of autobiographical remembering. A wealth of data now indicate that during episodic retrieval, a left-lateralized parietal Old/New effect^15,17,35–37^ that scales with perceived memory strength^9,17^ is present in the lateral IPS. This response profile has been found to shift in adjacent SPL and medial IPS toward signals that track uncertainty about recognition memory decisions^9,17^. Notably, our findings indicate that dorsal PPC Old/New effects are not selective to episodic memory decisions, but also extend to autobiographical semantic judgments. Interestingly, we did not observe a significant SPL uncertainty effect in our study, although the activity profile in SPL did exhibit the qualitative inverted-U profile associated with that effect. One possibility to be explored in future experiments is whether the temporally-extended nature of our memory probes results in a temporal blending of uncertainty and Old/New memory signals in SPL.

Interestingly, our left parietal findings also highlighted the apex of AnG (as opposed to lateral IPS)^10,15,46^ as a potential locus for a mnemonic evidence accumulator mechanism that incorporates autobiographical semantic memory. This dorsal component exhibited a significant graded pattern, with activity declining from autobiographical recollection to autobiographical semantic remembering to correct rejections. As such, we provide evidence that dorsal AnG contributes to the experience of autobiographical semantic remembering, while further increasing its response in cases where episodic remembering (F, and particularly R) occurs. This novel finding is consistent with evidence for AnG activation during non-autobiographical semantic retrieval tasks^40–42,68^. This finding is also complemented by evidence from our voxel-level analysis that bilateral ventral AnG exhibited a pure recollection effect, which may correspond to the long-standing association between AnG (broadly defined) and episodic memory retrieval^14,15,39^. Such graded signals in AnG may reflect a constructive process whereby event memory is built upon initial recognition of stimuli as being Own Life, as some participants reported (see Supplement). Taken together, our data highlight functional differentiation pertaining to autobiographical episodic and semantic decisions between dorsal and ventral lateral PPC, and within ventral lateral PPC.

It is also noteworthy that our voxel-level analyses revealed a continuous memory response profile within medial parietal cortex. Retrosplenial cortex/posterior cingulate cortex have been associated with recollection^12,18^, as well as familiarity^69–71^. Moreover, it has been suggested that PCC may be a critical hub for linking episodic and semantic information^40^. As such, integration or blending with autobiographical semantic memory information^1^, coupled with network-level connectivity^63^, could give rise to the shared response profile between this region and dorsal AnG supporting multiple levels or forms of autobiographical remembering.

Lastly, our exploratory analyses highlighted divergent memory response profiles within prefrontal cortex. It is now well-documented that subdivisions of lateral prefrontal cortex are associated with large-scale frontoparietal networks that underlie top-down attention (directing attention to goal-relevant information) and cognitive control (flexibly aligning cognitive and sensorimotor operations based on processing goals)^72–76^. These two networks respectively include the posterior superior frontal sulcus (SFS) and the inferior frontal sulcus (IFS), and these networks are characterized by differential intrinsic connectivity between (a) the SFS and SPL and medial IPS, and (b) IFS and lateral IPS^63^. Top-down attention and cognitive control are tightly coupled sets of processes that can influence not only what information we encode^77–84^, but also memory retrieval. Left components of the cognitive control network are prominently associated with retrieval success (i.e., old > new) effects^8,9,17,48^ and subdivisions of PCC and prefrontal cortex that share retrieval activity profiles exhibit a spatial organization that largely aligns with their intrinsic connectivity networks^9^.

Our data extend this evidence to real-world autobiographical memory retrieval. Episodic memory (R and F) retrieval were associated with prefrontal components of the top-down attention network – a response profile shared by the anterior-most parietal portions of this same network (but notably to the exclusion of SPL, which has been previously been shown to share a response profile with these frontal subdivisions that tracks memory uncertainty)^9^. By contrast, memory retrieval activity that did not differentiate between autobiographical episodic and autobiographical semantic memory was associated with the cognitive control network within prefrontal cortex (Fig. 5). This may reflect the more general association between IFS and Old/New memory effects^8,9,17,48^, and importantly extends such observations to memory for autobiographical semantic information.

The continuous response profile (as identified in our AnG ROI) was the dominant response profile in medial prefrontal cortex. Medial prefrontal cortex is associated with the default mode network^63^ – of which both medial prefrontal cortex and AnG are a part, and which has been associated with increasing activity from familiarity to recollection judgments in a Remember/Know paradigm^85^. Our data, however, indicate that this response profile is graded for autobiographical memory retrieval, decreasing from episodic familiarity to autobiographical semantic memory, and from autobiographical semantic memory to novel lures. Interestingly, a ventral component of medial prefrontal cortex was associated selectively with recollection in our paradigm, perhaps indicative of its robust anatomical connections with the anterior hippocampus^86^ and its role in hippocampally-mediated item-spatial context retrieval^87^. Although we did not collect resting state data in our experiment, these findings contribute to the growing literature that intrinsic functional organization of the brain underlies different computations related to memory decisions.

Collectively, our findings support a view in which processes in specific components of a broad “core recollection network”^12^ also contribute to or draw upon real-world autobiographical semantic remembering. Our findings revealed significant functional differentiation within the MTL, with anterior MTL cortex and hippocampus selectively supporting the experience of autobiographical episodic memory, while the hippocampal tail appears to contribute to a more general autobiographical memory retrieval mechanism that encompasses the experience of acontextual autobiographical semantic remembering. Our data indicate that most left lateral PPC subdivisions contribute to both autobiographical episodic and semantic remembering, but with divergent activity profiles. In particular, AnG may be a candidate for accumulating mnemonic evidence from autobiographical semantic remembering through different degrees of episodic memory strength. As such, our data offer novel insights that bear on theories of MTL and parietal cortex functional organization and elucidate circuitry that supports different forms of real-world autobiographical memory.

## Electronic supplementary material

Supplemental Information
