# Supplementary material for: Differential Medial Temporal Lobe and Parietal Cortical Contributions to Real-world Autobiographical Episodic and Autobiographical Semantic Memory
Source: Sci Rep. 2018 Apr 18;8:6190. doi: 10.1038/s41598-018-24549-y (PMC5906442; doi:10.1038/s41598-018-24549-y)
Supplement: Supplementary file 1 — Supplemental Information [file 41598_2018_24549_MOESM1_ESM.pdf]

Supplementary Information for

Differential Medial Temporal Lobe and Parietal Cortical Contributions to Real-world Autobiographical Episodic and Autobiographical Semantic Memory

Authors:
Thackery I. Brown[1]*, Jesse Rissman[2], Tiffany E. Chow[2], Melina R. Uncapher[3], Anthony D. Wagner[4,5]

Affiliations:
[1] School of Psychology, Georgia Institute of Technology, Atlanta, Georgia, United States of America,
[2] Department of Psychology, University of California, Los Angeles, Los Angeles, California, United States of America,
[3] Department of Neurology, University of California, San Francisco, San Francisco, California, United States of America,
[4] Department of Psychology, Stanford University, Stanford, California, United States of America,
[5] Stanford Neurosciences Institute, Stanford University, Stanford, California, United States of America

*Corresponding Author:
Thackery I. Brown (thackery.brown@psych.gatech.edu)
Memory, Affect, and Planning Laboratory
School of Psychology
Georgia Institute of Technology
654 Cherry Street NW
Atlanta, GA 30332

Pre-scan Memory Test Instructions

During the fMRI scanning session, we will assess your memory for selected experiences recorded by your Vicon Revue camera over the past month. On each trial, you will be presented with a sequence of four photographs that either constitute a brief 1-5 minute episode from your life or else were recorded by the camera of another participant in this study. Immediately after viewing each photo sequence, you will be prompted to select one of the eight possible response options to indicate your level of memory for that episode. The eight response options are described on the next page, but first we will review the distinction between the mnemonic experiences of *recollection*, *familiarity*, and *knowing*.

Understanding the distinction between Recollection, Familiarity, and Knowing:

On some trials, you will have moderate or high confidence that the presented sequence of images depicts an experience from your life. Regardless of your level of confidence, we ask that you reflect on whether your memory retrieval experience includes the **recollection** of specific details associated with that particular moment of your past, or whether the depicted event feels **familiar** to you even though you don't recollect any specific details of the event, or you **know** that that event must have come from your camera but you don't recognize it as familiar and you don't recollect any details. The distinction between *recollection, familiarity,* and *knowing* is clarified below.

Recollection is the ability to become consciously aware again of some aspect or aspects of what happened or what you experienced at the specific moment of your life. For example, the photographs may remind you of where you were, what you were doing, what you were thinking, or what you were feeling during that brief episode of your life. You should only make a ***Recollected*** response when you are able to recollect details associated with the particular life experience beyond those that are evident in the photos themselves. Please try to avoid confusing a strong sense of familiarity with the experience of recollection.

Examples of recollection-based memories:

"I recollect that moment in my economics class because the professor made a really cheesy joke."

"I recollect that particular bike ride across campus because I was running late to meet some friends for lunch".

"I recollect that visit to the cafeteria because I recall being disappointed in the food offerings that day".

Familiarity is the feeling that you get when the images remind you in some way of this moment from your past. You likely have experiences of familiarity without recollection frequently, such as seeing another student's face and having a moderate or strong sense that you've seen the individual before, even though you can't recollect any details about your past encounter. You likely will have this type of experience when viewing some of the photo sequences in this experiment. The examples below illustrate the common phenomenon of familiarity-based recognition that is unaccompanied by recollection. When you experience this type of memory, you should make a ***Familiar*** response.

Examples of familiarity-based memories:

"Seeing that photo of the woman in the bright pink dress walking in front of me on campus definitely triggered a strong sense of familiarity, but I don't recollect anything about that particular experience, such as where I was heading or what I was thinking about at that time".

"That moment in class seems pretty familiar to me, but I can't recall when that was or what topics were being discussed or any other specific details".

"That experience of bumping into my roommate in Tressider feels very familiar, but to be honest, I really can't recollect when that was or anything about our conversation or the broader context (beyond the fact that I was clearly chatting with him/her in Tressider)."

Knowing refers to the recognition that the photos must have been taken by your camera, even when they evoke little or no familiarity or recollection for that moment of your life. Simply recognizing that the photos were

taken in a particular location, include familiar people (e.g. friends, classmates, co-workers, or professors), or include familiar objects (e.g., your laptop, cell phone, water bottle, bike, or car) is NOT SUFFICIENT to warrant a *Familiar* response. You should only make a *Familiar* response if some elements of that particular experience feel familiar you. If something in the photos gives away the fact that this must have been your experience, but fails to trigger any feelings of familiarity for that experience, you should make the ***Know but Not Familiar*** response.

THE 8 RESPONSE OPTIONS

(in the scanner you will learn to map these 8 possible responses onto 8 different buttons)

SURE NOT YOURS: You are sure that you did not have this experience

Select this response if you are highly confident that this was someone else's experience.

PROBABLY NOT YOURS: You probably did not have this experience

Select this response if you suspect that this was someone else's experience.

UNSURE: You are unsure whether or not this was your experience

Select this response if you cannot discern whether this was your experience, or if you have a very vague sense that this was your experience. Note that your uncertainty about whether this was your experience may stem from the fact that all of the participants in this study were Stanford students, and thus you will recognize many of the places and entities depicted in other participants' photos.

KNOW BUT NOT FAMILIAR: You know that this was your experience, and yet the specific experience depicted in the photos does not seem particularly familiar to you

Select this response when the depicted specific event fails to elicit a sense of familiarity, and yet you notice that the photos include details that indicate that they must have been captured by your camera. Please be careful not to confuse your familiarity for the people, places, or things in the photos with your familiarity (or lack thereof) for this specific moment of your life.

MODERATELY FAMILIAR: This specific experience seems moderately familiar to you

Select this response when the depicted specific event elicits a moderate feeling of familiarity, but does not evoke recollection of any of the details surrounding the event (i.e., viewing these photos does not "take you back" to this specific moment of your life).

STRONGLY FAMILIAR: This specific experience seems strongly familiar to you

Select this response when the depicted specific event elicits a strong feeling of familiarity, yet fails to evoke recollection (i.e., viewing these photos does not "take you back" to this specific moment of your life).

MODERATELY RECOLLECTED: You are able to recollect a few of the details surrounding this specific experience

Select this response when the depicted specific event elicits recollection of a few of the details surrounding this specific moment of your life (e.g., one or a few specific details about this specific experience, beyond those present in the photos themselves, come to mind).

STRONGLY RECOLLECTED: You are able to recollect many details this specific experience

Select this response when the depicted specific event elicits strong recollection of this specific moment of your life (e.g., viewing these photos "takes you back" to this specific moment of your life and you vividly recall many specific thoughts, feelings, and/or contextual details about this specific experience).

Post-scan interview reports

Following Experiment 1, participants were given an extensive post-scan interview. Although the majority of questions in our post-scan interview were not relevant to the present study, responses to three questions give added insight into how participants approached the memory judgments in the task:

1) When asked "*Which kinds of image features were most useful for you when making memory judgments?*" – The majority of participants indicated recognition of people, with many also indicating places and/or things. One example participant's description of which image features were "most useful": "Bike handles, laptops, teachers, friends". These are the type of personal semantic details that underlie Know memory, and although their recognition was sufficient for an Own Life judgment, per our instructions they were not sufficient to warrant a "Familiar" or "Recollection" response.

2) When asked "*What types of strategies did you use to arrive at your memory ratings?*" - All participants reported reported looking for image recognition indicative of autobiographical semantic knowledge (e.g., "I looked for familiar faces/surroundings" and "seeing if I recognized what was in the picture and the people"). Many described this as a multi-step process, wherein after they determined that the photo sequence was from their life, they then tried to remember the event or assessed its familiarity. One example strategy description: "I first tried to see if the pictures were mine, and then if it was, I tried to see if I can come up with additional details".

3) When asked "*When you made 'Know but Not Familiar' or 'Familiar' responses, how often did you find yourself recollecting extraneous details from your past (i.e., details other than those associated with that particular moment of your life)?*" – All but three participants indicated "never" or "not very often". One participant explicitly ventured "when I did 'know but not familiar', I tried to think hard to make sure I wasn't missing anything". When coupled with the fact that participants exhibited extremely high discriminability for Know responses (corrected d' = 1.96), these reports suggest that fMRI activity for Know responses was not colored by the intrusion of episodic memory for other life events or general recognition of a familiar place from another's life.

Overall, participant responses to these interview questions suggest that autobiographical semantics were an important factor for participants' memory decisions.
